# Building a predictive model for polycyclic aromatic hydrocarbon dosimetry in organotypically cultured human bronchial epithelial cells using benzo[*a*]pyrene

**DOI:** 10.1016/j.toxrep.2025.102133

**Published:** 2025-09-28

**Authors:** Victoria C. Colvin, Kelley M. Bastin, Lisbeth K. Siddens, Monica L. Vermillion Maier, David E. Williams, Jordan N. Smith, Susan C. Tilton

**Affiliations:** a Department of Environmental and Molecular Toxicology, Oregon State University, Corvallis, OR, USA; b OSU/PNNL Superfund Research Program, Oregon State University, Corvallis, OR, USA; c Pacific Northwest National Laboratory, Richland, WA, USA

**Keywords:** Polycyclic Aromatic Hydrocarbons, Benzo[*a*]pyrene, Dosimetry Modeling, Human Bronchial Epithelial Cells, Metabolizing Enzymes

## Abstract

The airway epithelium is a primary route of exposure for inhaled toxicants, and organotypic culture models represent an important advancement for toxicity testing compared to simple *in vitro* models that may lack metabolic capability and multicellular structure/communication associated with the bronchial epithelium *in vivo*. A quantitative understanding of chemical dosimetry is key for interpreting and extrapolating study results; however, dosimetry is understudied in organotypic models limiting ability to predict toxicity. We developed a dosimetry model for primary human bronchial epithelial cells (HBECs) cultured at the air-liquid interface (ALI) using benzo[*a*]pyrene (BAP), a representative polycyclic aromatic hydrocarbon. Dose and time course evaluation of metabolite formation and enzyme activity and expression were utilized to parameterize a cellular dosimetry model to improve the utility of ALI-HBECs for assessing chemical risk. Dosimetry analysis demonstrated absorption of BAP into cells and an increase in Phase 1 and 2 metabolites over time that correlated with regulation of metabolizing enzymes. BAP was cleared from cells by 48 h after exposure, and the primary metabolites generated in ALI-HBECs were BAP-3-phenol, BAP-4,5-dihydrodiol, BAP-7,8-dihydrodiol, BAP-9,10-dihydrodiol, BAP-7,8,9,10-tetrol, BAP-3-phenol-glucuronide, BAP-4,5-dihydrodiol-glucuronide, and BAP-9,10-dihydrodiol-glucuronide. The resulting dosimetry model described BAP and 7,8-dihydrodiol toxicokinetics in ALI-HBECs and suggested active excretion of 7,8-dihydrodiol. Overall, this study demonstrates metabolic competency of ALI-HBECs for BAP metabolism, demonstrates the usefulness of complex *in vitro* systems for human-relevant toxicity data, and exhibits how *in silico* models can be utilized for understanding the dosimetry of test compounds to aid in *in vitro* to human extrapolation of toxicity data for risk assessments.

## Highlights

• Human bronchial epithelial cells formed known Phase 1& 2 benzo[*a*]pyrene metabolites.
• A cellular dosimetry model accurately predicts benzo[*a*]pyrene toxicokinetics.
• The model suggests metabolism induction and active excretion of metabolites.

## Graphical Abstract

**Figure:**
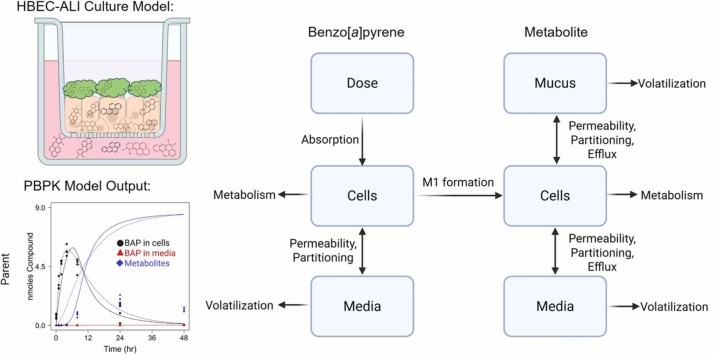

## 1 Introduction

New approach methodologies (NAMs) including complex *in vitro* systems and *in silico* predictive models are being rapidly developed to improve chemical safety research [94]. NAMs are essential tools in advancing the Tox21 initiative of developing high throughput *in vitro* and *in silico* test systems for acquiring more human-relevant chemical safety data while reducing the load on animal models for human health and toxicity assessments [11], [65], [94], [105]. However, their acceptance and use in regulatory risk assessments has been slow and limited due to the need for validation of the systems for compounds of interest before gaining confidence in their inclusion for risk assessments [11], [65], [86], [94].

Several complex *in vitro* systems such as organotypic or 3D cultures of human cells have been developed for multiple human organ systems to improve upon traditional 2D culture or *in vivo* models for collecting high throughput and human-relevant toxicity data [49], [95]. For example, human lung epithelial cells cultured at the air-liquid interface (ALI) recapitulate the *in vivo* human lung with great accuracy in cellular differentiation, morphology of the epithelium, and constitutive levels of global gene expression and have been used for toxicity assessment of inhaled compounds [5], [10], [20], [39], [51], [58], [82], [83], [106]. The acceptance of these ALI models by regulatory agencies is contingent on improving their utility to provide accurate and relevant toxicity data for human health risk assessment. A key step in this process is understanding the dosimetry of chemical treatments applied to the culture system and is critical to support quantitative methods such as *in vitro* to *in vivo* extrapolation (IVIVE) [10], [29], [57], [63], [104], [119]. The development of predictive dosimetry models for *in vitro* test systems will aid our understanding of compound interactions with test systems and will further support their use as NAMs and utilization for IVIVE [22], [57], [63], [86], [88], [91]. While *in silico* NAMs are becoming more available and proving to be very useful, many are based on *in vivo* animal data limiting their applicability to humans and adding extra uncertainty factors when conducting IVIVE [63]. Therefore, there is a need to develop more human-relevant *in silico* NAMs that can assist in both understanding dosimetry of *in vitro* systems and predicting potential toxic effects from compounds of interest. Presently, we explore the applicability of toxicity data generated from ALI cultured human lung cells for constructing a cellular dosimetry model to describe and predict the dosimetry of polycyclic aromatic hydrocarbons (PAHs).

PAHs are a large class of chemicals consisting of two or more fused aromatic rings [56], [59]. They are ubiquitous in the environment from natural and anthropogenic sources of incomplete organic material combustion such as vehicle emissions, wildfires, and tobacco smoking [60], [68], [72], [78], [116], [118], [121]. Humans are exposed to PAHs through inhalation, ingestion, and dermal routes with the lung being a major target organ from inhaled exposures. PAHs have been shown to exert toxicity by increased carcinogenicity, genotoxicity, mutagenicity, and reduced overall lung function [2], [3], [13], [15], [16], [18], [36], [56], [71], [74], [84], [113], [116]. The US EPA has identified several PAHs as priority pollutants due to their potential for human health concerns, and benzo[*a*]pyrene (BAP) is often used as a representative for the class of PAHs for its high carcinogenic potential and overall toxicity [15], [17], [24], [50], [56], [62], [71], [84], [108], [113], [116]. Since PAHs often require metabolic activation in order to exert toxicity, and in particular carcinogenicity, it is important for them to be evaluated in a system with the capacity to metabolize them so their toxicity is not underestimated [1], [2], [3], [6], [12], [17], [43], [53], [71], [90], [107]. ALI cultured human lung cells have been shown to express many genes related to xenobiotic metabolism and retain the inducibility of several xenobiotic metabolizing enzymes like CYP1A1, but their capacity to metabolize PAHs into specific toxic intermediates and the dosimetry of treated PAHs in the system is not yet fully understood [8], [12], [23], [24], [41], [47], [69], [70], [76], [87], [89].

In this study, we developed a dosimetry model for PAHs in ALI cultured primary human bronchial epithelial cells (ALI-HBECs) using BAP as a reference compound. We assessed ALI-HBEC capacity for PAH metabolic activation, formation of PAH reactive intermediates, and dosimetry of treated PAH and applied the collected data to a cellular dosimetry model for predicting PAH dosimetry and metabolism in ALI-HBECs. Overall, this study validates ALI-HBEC competency for PAH metabolism, describes PAH interactions with ALI-HBECs through a dosimetry model, and adds confidence to the power of *in vitro* and *in silico* NAMs for providing human-relevant, accurate, and predictive data for IVIVE and human health risk assessments.

## 2 Methods

### 2.1 Chemicals and reagents

Benzo[*a*]pyrene (BAP, CAS# 50–32–8), BAP-7,8,9,10-tetrol (CAS# 61490–66–2), BAP-9,10-dihydrodiol (CAS# 57303–98–7), BAP-4,5-dihydrodiol (CAS# 57304–00–4), BAP-7,8-dihydrodiol (CAS# 57303–99–8), 9-phenol BAP (CAS# 17573–21–6), BAP-3-phenol (CAS# 13345–21–6), and dibenzo[def,*p*]chrysene (DBC, CAS# 191–30–0) were purchased from the Oregon State University Superfund Program PAH Repository (http://limsweb.science.oregonstate.edu). PneumaCult™-Ex Plus medium and PneumaCult™-ALI medium were purchased from STEMCELL Technologies, Vancouver, Canada. β-glucuronidase from limpets was purchased from MilliporeSigma, St Louis, MO.

### 2.2 Cell culture

Normal HBECs (Lonza, Basel, Switzerland) were expanded to passage 4 in PneumaCult™-Ex Plus medium and transferred to 12-well plates with 12 mm transwell inserts (STEMCELL Technologies, Vancouver, BC, Canada) and 30,000 cells per insert or 24-well plates with 6.5 mm transwell inserts (STEMCELL Technologies, Vancouver, BC, Canada) and 9000 cells per insert for differentiation at the ALI using PneumaCult™-ALI medium. Cells were cultured at the ALI for 25 days at 37°C and 5 % CO_2_ with media changes every 2–3 days and mucus washes using DPBS on the apical surface once a week starting at 14 days post air-lifting to maintain healthy ALI cultures. Just before exposure, media was changed and ALI-HBECs were apically washed with DPBS or ALI media to remove mucus.

### 2.3 Mass balance experiments

To assess PAH dosimetry and metabolism over time, ALI-HBECs cultured in 12-well plates were treated with BAP (2.26 µg/cm^2^) in ALI media with 1 % DMSO vehicle and evaluated for Phase 1 metabolite formation at 0, 1, 2, 4, 8, 24, and 48 h. These data were utilized for optimizing the dosimetry model parameters. To validate the dosimetry model and further evaluate PAH metabolism over time, ALI-HBECs cultured in 12-well plates were treated with BAP (4.67 µg/cm^2^) in ALI media with 1 % DMSO vehicle and evaluated for Phase 1 and 2 metabolite formation at 0, 8, 24, and 48 h. Glass pipettes were used for all collection and extraction steps to reduce loss of compound from binding to plastic. ALI media was used instead of DPBS for all rinse and collection steps due to interference with the fluorescence detector. At each timepoint, basal media was collected and the bottom of the insert rinsed, the apical surface was washed and rinses collected, and cells were collected by scraping with a stainless-steel spatula and rinsed from the insert. All collected fractions were flash frozen in liquid nitrogen and stored at −80°C.

### 2.4 Analytical methods

We used liquid-liquid extraction and liquid chromatography to measure BAP and Phase 1 metabolites in samples. For Phase 2 metabolite analysis, samples were incubated with β-glucuronidase to deconjugate glucuronide and sulfide metabolites following methods described by Combie et al. [30]. For cell fractions, 0.1 mm glass beads were added to the sample and placed in a Next Advance Bullet Blender™ 24 (Troy, NY) for 3 min to fully lyse cells and increase recovery. All cell, media, and mucus samples were transferred to amber glass vials and adjusted to pH 5 with acetic acid. β-glucuronidase reconstituted in water was added to samples at 5000 U/mL, and samples were incubated at 65°C for 3 h. Samples were allowed to cool to room temperature before continuing with extractions.

All samples were spiked with DBC as an internal standard for a final concentration of 2.5 μM to correct compound recovery for extraction efficiency. Cell fractions not previously incubated with β-glucuronidase were lysed by blending with 0.1 mm glass beads. All cell, media, and mucus samples were transferred to glass vials, vortexed and sonicated in ethyl acetate, and incubated for 2 h at −20°C to precipitate any protein. Samples were centrifuged at 2000 rcf for 10 min, and the ethyl acetate supernatant was collected. This was repeated for a total of three rounds. Ethyl acetate was evaporated to dryness under nitrogen gas, samples were reconstituted in 100 μL ACN, and samples were stored at −80°C.

Samples were analyzed for BAP parent and six Phase 1 metabolites by Ultra-Performance Liquid Chromatography (UPLC) using a Waters Acquity H-Class System (Milford, MA) as described in Vermillion-Maier et al. [109]. Briefly, separation of compounds was achieved with a Waters Acquity UPLC BEH C 18 1.7 μm 2.1 × 50 mm column (Milford, MA) with a Vanguard HSS PFP 1.8 μm 2.1 × 5 mm guard column (Milford, MA) using solvents labelled as (A) 0.3 % formic acid in water and (B) PEG-free ACN. Compounds were eluted over 20 min at 0.25 mL/minute under a gradient of 0.0 min 70:30 A:B; 0.1 60:40; 10.0 54:46; 10.1 43:57; 14.3–16.3 0:100; 16.4–20 min 70:30, and eluted compounds were detected by a Waters Acquity 2475 Fluorescence (FLR) Detector (Milford, MA). Elution times were identified for BAP parent, BAP-7,8,9,10-tetrol, BAP-9,10-dihydrodiol, BAP-4,5-dihydrodiol, BAP-7,8-dihydrodiol, BAP-9-phenol, BAP-3-phenol, and DBC (Table 1) by analytical standards and quantified by comparing to a seven-point calibration curve. The percent BAP recovered at each timepoint was calculated as the amount of BAP parent (Eq 1 A) or the amount of BAP parent and metabolites (Eq 1B) summed over cell, media, and mucus fractions in a BAP treated sample divided by the amount of BAP added to the system during treatment estimated by the amount of BAP recovered in samples collected from 0-hour exposures.

(1A) $$\%{\mathrm{BAP}}_{\mathrm{parent}}=\frac{\sum_{\mathrm{cell},\mathrm{media},\mathrm{mucus}}{\mathrm{BAP}}_{m}}{{\mathrm{BAP}}_{i}}$$

(1B) $$\%{\mathrm{BAP}}_{\mathrm{total}}=\frac{\sum_{\mathrm{cell},\mathrm{media},\mathrm{mucus}}{[\mathrm{BAP}}_{m}+\sum_{1}^{6}{\mathrm{Met}}_{x,m}]}{{\mathrm{BAP}}_{i}}$$

**Table 1:** UPLC elution times of BAP, Phase 1 metabolites, and DBC internal standard.

**Compound**	**Elution Time (minutes)**
BAP−7,8,9,10-tetrol (1)	1.5
BAP−7,8,9,10-tetrol (2)	2.2
BAP−9,10-dihydrodiol	2.7
BAP−4,5-dihydrodiol	4.3
BAP−7,8-dihydrodiol	4.6
BAP−9-phenol	12.7
BAP−3-phenol	13.1
BAP	15.1
DBC	16.0

where *BAP*_*m*_ is the measured amount of BAP parent in one fraction, *BAP*_*i*_ is the estimated amount of BAP initially added to the system during treatment from 0-hour samples, and *Met*_*x,m*_ is the measured amount of metabolite x in one fraction for each of the six BAP Phase 1 metabolites. Glucuronide metabolite formation was estimated as the difference between samples incubated with and without β-glucuronidase at similar timepoints.

### 2.5 RNA-sequencing

To assess ALI-HBEC constitutive and differential gene expression, ALI-HBECs cultured in 24-well plates were apically treated with BAP (0.076, 0.38, 1.5, or 3 µg/cm^2^) in DPBS with 1 % DMSO vehicle or treated with only a DPBS with 1 % DMSO vehicle. Cells were collected from inserts and lysed with RLT lysis buffer with 1 % β-mercaptoethanol using the RNeasy Mini Kit (Qiagen, Hilden, Germany) and stored at −80 °C. RNA was isolated from cell lysate using the RNeasy Mini Kit per the manufacturer’s protocol and was quantified on a Synergy HTX (BioTek, Winooski, VT, USA) plate reader equipped with a Take3 module (BioTek, Winooski, VT, USA). RNA integrity was evaluated using an Agilent Bioanalyzer (Agilent, Santa Clara, CA) with samples having values ≥ 8.0 used for library construction and paired-end sequencing on a DNBSEQ platform at the Beijing Genomic Institute (BGI) Americas (www.bgi.com). For library construction, mRNA was poly-A selected and libraries were prepared as previously described by Dasgupta et al. [35]. High quality reads with a sequencing length greater than 150 bp were annotated and mapped to the human reference genome GRCh38.p14 using HISAT2, count tables were generated with feature Counts, and differential expression was conducted using the DESeq2 R package version 1.46.0 compared to vehicle control [54], [64]. In vehicle exposed samples, the fragment per kilobase million (FPKM) was calculated for each gene, and genes with an FPKM > 1 were considered constitutively expressed [48], [102], [114]. Genes associated with xenobiotic metabolism and transport were identified by pathway enrichment analysis in MetaCore (GeneGo, Thomson Reuters, Carlsbad, CA) and by literature search.

### 2.6 CYP1A1 activity and cell viability

To assess changes in CYP1A1 activity over time after PAH exposure, ALI-HBECs cultured in 24-well plates were apically treated with BAP (2.26 µg/cm^2^) in a DPBS with 1 % DMSO vehicle for 0, 2, 4, 6, 8, 24, or 48 h. CYP1A1 activity was measured in ALI-HBECs using the P450-Glo™ CYP1A1 Assay System (Promega, Madison, WI). The protocol was adapted to assess CYP1A1 activity in ALI-HBECs. Luciferin-CEE solution was diluted in DPBS, ALI-HBEC basal media was replaced with DPBS, and 50 µL of Luciferin-CEE solution was added to the apical surface. After a 3 hr incubation at 37°C, 25 µL of apical liquid was transferred to a white 96-well plate (Thermo Fisher Scientific, Waltham, MA), and 25 µL of detection reagent was added to every well. Luminescence was recorded on a Synergy HTX plate reader. Cell viability was measured using the CellTiter-Glo® (CTG) Luminescent Cell Viability Assay (Promega, Madison, WI) per manufacturer’s protocol, and luminescence was recorded on a Synergy HTX plate reader. Background luminescence was subtracted from all data, and CYP1A1 activity was normalized to cell viability for each sample. Results were transformed to percent vehicle by dividing by their respective vehicle controls.

### 2.7 Cytotoxicity

To evaluate if metabolite formation and movement could be affected by loss of cell viability, lactate dehydrogenase (LDH) leakage was measured in the basal media. After exposure for metabolite analysis, 50 μL of basal media was collected and evaluated for cytotoxicity by LDH leakage using the CyQUANT™ LDH Cytotoxicity Assay (Invitrogen, Thermo Fisher Scientific, Waltham, MA) per manufacturer’s protocol. Cytotoxicity was calculated by subtracting absorbance at 680 nm from absorbance at 490 nm using a Synergy HTX plate reader. Results were transformed to percent vehicle by dividing by their respective vehicle controls.

### 2.8 Barrier integrity

To evaluate if metabolite formation and movement could be affected by decreased barrier integrity, transepithelial barrier integrity (TEER) was measured using an epithelial volt-ohmmeter (World Precision Instruments, Sarasota, FL). The volt-ohmmeter was calibrated using a test electrode prior to all measurements. Prior to cell collection for metabolite analysis, fresh ALI media was added to the apical and basal chambers, and resistance was measured (ohms) for each insert. Results were adjusted for background resistance by subtracting resistance measured from an insert without cells. Results were transformed to percent vehicle by dividing by their respective controls.

### 2.9 Statistical analysis

RNA-seq, cytotoxicity, and barrier integrity endpoints had 4 replicates per treatment and timepoint. The optimization UPLC dataset had 4 replicates per treatment and timepoint. The validation UPLC dataset had 4 replicates per treatment at 0 h and 8 replicates per treatment at 8, 24, and 48 h where 4 replicates per treatment were incubated with β-glucuronidase prior to extraction. Statistical analyses were conducted in GraphPad Prism 10 version 10.4.1 (Dotmatics, Boston, MA) by one-way ANOVA with Dunnett’s post-hoc test for all BAP treatments or *t*-test for positive control treatments. A significance level cutoff was defined at an adjusted p-value of 0.05.

### 2.10 ALI-HBEC PAH dosimetry model

**Fig. 1:**
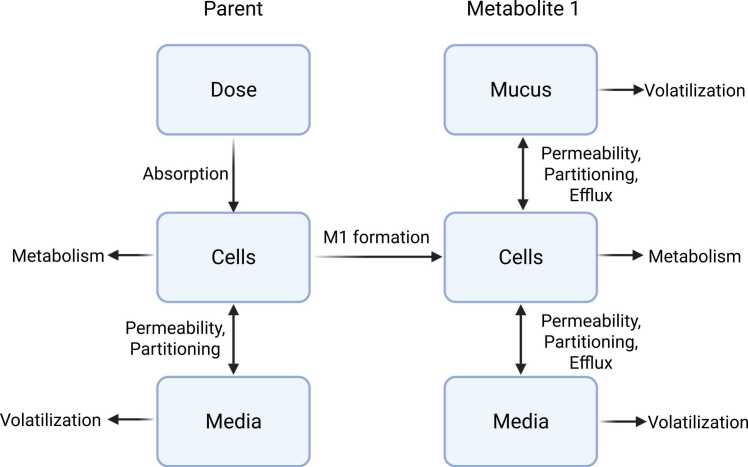
Compartmentalized cellular dosimetry model for a PAH parent and metabolite in ALI-HBECs.

A dosimetry model was developed to contextualize how PAHs interact with ALI-HBECs during exposure (Fig. 1). The model describes the time course dosimetry of two PAHs: BAP, the parent PAH, and BAP-7,8-dihydrodiol, a precursor metabolite to the primary ultimate toxicant (BPDE). Model parameters for cell and media compartments were characterized based on measurements for the ALI-HBEC culture system described by STEMCELL Technologies (Table 2) [100]. For simplicity, the model does not include parent partitioning between mucus and cell compartments and instead models PAH absorption from a dose compartment as a first order rate. The formation of metabolite 1 (M1) was modeled as a fraction of total parent metabolite formation. Mucus production was estimated based on normal human lung mucus production consisting of 96 % water and 4 % solid content with a secretion of 4 µL/cm^2^*h from data reported by Clarke and Parvia and Martens et al. [28], [67]. We initially modeled parent and metabolite transport among compartments as passive diffusion using ordinary differential equations. To improve model fit for 7,8-dihydrodiol distribution, we included an active efflux of the metabolite from cells to media and mucus. BAP elimination was described through metabolism in cells or volatilization from the media. 7,8-dihydrodiol elimination was described through metabolism in cells or volatilization from the media and mucus. We modeled parent and metabolite metabolism in the lung cells using clearance terms scaled by the estimated volume of cells to the ¾ power as is recommended by the US EPA [42], [96], [112].

**Table 2:** Constant physiological parameter values used for dosimetry modeling.

**Parameter**	**Value**
Volume of basal media	1 mL
Surface area of transwell	1.12 cm^2^
Height of cells	∼35 µm*
Volume of cells	0.00392 cm^2^
Rate of mucus secretion	0.004 mL/cm^2^*hr^a^
BAP cell:media partitioning	110
7,8-dihydrodiol cell:media partitioning	73
7,8-dihydrodiol cell:mucus partitioning	10.4
Metabolism Induction Coefficient A	6.55
Metabolism Induction Coefficient B	1.17
Metabolism Induction Coefficient C	13.00
Metabolism Induction Coefficient D	23.29
Metabolism Induction Coefficient E	373.05
Metabolism Induction Coefficient F	2032.59

* STEMCELL Technologies [100]

a Martens et al. [67]

Researchers have shown that exposure to BAP and other PAHs induces CYP enzymes that in turn metabolize the PAH [101]. Consistent with these previous observations, we observed induction of CYP1A1 activity as measured by P450-Glo™ (see Results). We included a description of induced metabolism to accurately recapitulate observed concentrations of BAP caused by increased metabolism as the experiment progressed. We scaled PAH metabolism to match measured CYP1A1 activity on a relative scale from 0 to 1, where a value of 1 is the highest observed activity in the data set, using a rational function (Eq. 2),

(2) $$\mathrm{CYP}=\;\frac{A*t^{2}+B*t+C}{D*t^{2}-E*t+F}$$

where *A – F* are coefficients (Table 2). CYP1A1 activity was included as a coefficient of the rate of metabolism for PAH parent which could be triggered by an induction switch. The switch allows for metabolism induction to be turned on or off in future uses of the model. When turned off, the *CYP* term is set to 1 and the rate of metabolism behaves as described above.

The BAP and 7,8-dihydrodiol cell:media partition coefficients and the 7,8-dihydrodiol cell:mucus partition coefficient were estimated according to methods described by Poulin and Theil [85]. Water, neutral lipid, and phospholipid content of lung cells were assumed to be similar to mouse lungs reported by Poulin and Theil [85]. The contents of PneumaCult™-ALI medium are proprietary. Therefore, water, neutral lipid, and phospholipid content of media was estimated by 10 % fetal bovine serum (FBS) in basal media based on lipid concentrations in 100 % FBS as reported by Newsom and Sullivan [77]. The water, neutral lipid, and phospholipid content of mucus was estimated by data reported by Woodward et al. [115].

A local sensitivity analysis was performed on the dosimetry model to identify the importance of parameters for simulating PAH parent and M1 concentrations in the cell, media, and mucus compartments to aid in model optimization. Analyses were conducted based on the measured amount of BAP added to the system for the optimization UPLC dataset, and sensitivity coefficients were calculated based on a 1 % change in the parameter with all other parameters held fixed. Maximum parameter sensitivity was classified according to brackets set by Teeguarden et al. (Table 3, Table 4) [103].

**Table 3:** Maximum sensitivity coefficients for dosimetry model parent parameters evaluated for influence on the amount of PAH parent in the cell and media compartments.

**Parameter**	**No Metabolism Induction**	**Sensitivity Classification**^a^	**With Metabolism Induction**	**Sensitivity Classification**^a^
**PAH parent in lung cells**				
Absorption into cells	1.00	H	1.00	H
Permeability of cells	7.97e−3	L	7.96e−3	L
Clearance by metabolism	3.39	H	3.31	H
Volatilization from media	1.04e−5	L	8.50e−6	L
**PAH parent in media**				
Absorption into cells	1.00	H	1.00	H
Permeability of cells	1.00	H	1.00	H
Clearance by metabolism	0.89	H	0.74	H
Volatilization from media	0.15	M	0.067	L

a Sensitivity classifications of low (L, less than 0.15), medium (M, 0.15 – 0.5), and high (H, greater than 0.5) based on [103].

**Table 4:** Maximum sensitivity coefficients for dosimetry model M1 parameters evaluated for influence on the amount of M1 in the cell, media, and mucus compartments.

**Parameter**	**Passive Diffusion**	**Sensitivity Classification**^a^	**Active Efflux**	**Sensitivity Classification**^a^
**M1 in lung cells**				
Fraction of metabolites that are M1	1.00	H	1.00	H
Permeability of cells	0.37	M	0.042	L
Clearance by metabolism	0.79	H	0.083	L
Volatilization from media	0.044	L	2.32e−3	L
Volatilization from mucus	1.32e−5	L	1.13e−4	L
Efflux to media	NA	NA	0.94	H
Efflux to mucus	NA	NA	0.50	H
**M1 in media**				
Fraction of metabolites that are M1	1.00	H	1.00	H
Permeability of cells	1.00	H	1.43e−3	L
Clearance by metabolism	0.59	H	0.050	L
Volatilization from media	0.050	L	0.060	L
Volatilization from mucus	5.46e−6	L	1.04e−6	L
Efflux to media	NA	NA	1.00	H
Efflux to mucus	NA	NA	0.31	M
**M1 in mucus**				
Fraction of metabolites that are M1	1.00	H	1.00	H
Permeability of cells	0.97	H	0.019	L
Clearance by metabolism	0.76	H	0.051	L
Volatilization from media	0.039	L	4.99e−5	L
Volatilization from mucus	5.39e−3	L	0.059	L
Efflux to media	NA	NA	0.59	H
Efflux to mucus	NA	NA	0.98	H

a Sensitivity classifications of low (L, less than 0.15), medium (M, 0.15 – 0.5), and high (H, greater than 0.5) based on [103].

Parameter optimization was prioritized based on their sensitivity classification for parent or M1 amount in cell, media, and mucus compartments with greater priority given to those with a high sensitivity classification (Table 5, Table 6). Parameters were optimized against the optimization UPLC dataset. Parent absorption and metabolism were optimized together against data for BAP in cells. Parent permeability and volatilization were optimized together against data for BAP in media. Due to low sensitivity, M1 volatilization from media and mucus was set as the same value for parent volatilization from media. For the passive diffusion model, M1 formation, permeability, and metabolism were optimized together against data for 7,8-dihydrodiol in cells and media and total amount of 7,8,9,10-tetrol. For the active efflux model, M1 permeability was set as the same value for parent permeability. M1 formation was optimized against data for 7,8-dihydrodiol in cells, media, and mucus. M1 metabolism was optimized against data for the total amount of 7,8,9,10-tetrol. M1 active efflux into media was optimized against data for 7,8-dihydrodiol in media. M1 active efflux into mucus was optimized against data for 7,8-dihydrodiol in mucus. All model parameters and CYP1A1 rational function coefficients were optimized using a maximum log likelihood objective with the Nelder-Mead algorithm and starting values set by visual inspection.

**Table 5:** Parent parameter values used for dosimetry model with or without metabolism induction.

**Parameter**	**No Metabolism Induction**	**With Metabolism Induction**	**Units**
Absorption into cells	0.47	0.24	hr^−1^
Volatilization from media	4.36e−3	1.85e−3	hr^−1^
Permeability of cells	6.93e−5	8.02e−5	cm/hr
Clearance by metabolism	4.08	7.95	L/hr/kg^3/4^

**Table 6:** M1 parameter values used for dosimetry model with passive diffusion or active efflux out of the cell compartment.

**Parameter**	**Passive Diffusion**	**Active Efflux**	**Units**
Fraction of parent metabolites that are M1	0.022	0.099	NA
Volatilization from media	1.85e−3	1.85e−3	hr^−1^
Volatilization from mucus	1.85e−3	1.85e−3	hr^−1^
Permeability of cells	0.061	8.02e−5	cm/hr
Clearance by metabolism	3.93	0.79	L/hr/kg^3/4^
Active efflux into media	NA	8.69e−4	L/hr
Active efflux into mucus	NA	4.45e−4	L/hr

The model was built in R version 4.4.2 (R Foundation for Statistical Computing, Vienna, Austria) using the RMagnolia R package version 1.3.15 (https://www.magnoliasci.com/rmagnolia/). The Akaike Information Criterion (AIC) was used for selecting the best model fit for CYP1A1 activity, inclusion of metabolism induction, and inclusion of active efflux. The final model with metabolism induction and active efflux of the metabolite was validated by comparison to data collected in the validation UPLC dataset.

## 3 Results

The overall goal of this study was to build a PAH dosimetry model in ALI-HBECs. BAP was chosen as a reference PAH due to knowledge about existing metabolism and toxicity in lung, as well as the availability of metabolite standards for quantification. BAP metabolite formation and distribution of BAP parent and metabolites in the culture system were assessed by UPLC with fluorescence detection and results compared to Phase 1 and 2 metabolizing enzyme mRNA expression and protein activity. Results were used to inform and optimize a dosimetry model of PAH parent and metabolite movement in ALI-HBECs over time, and the model parameters were evaluated to understand the kinetics of BAP movement and metabolism in ALI-HBECs.

### 3.1 Dosimetry of BAP and metabolites by UPLC

**Fig. 2:**
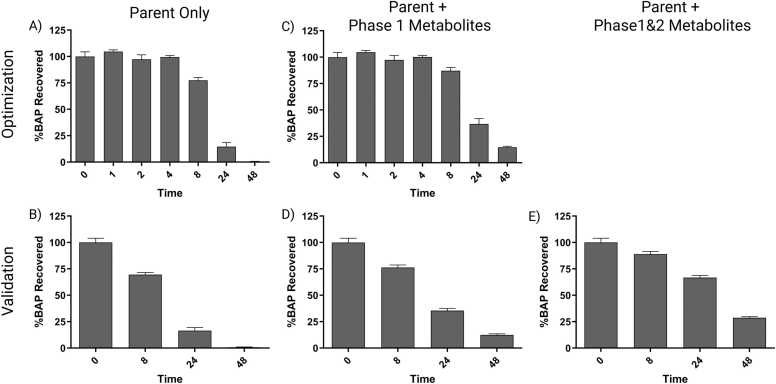
Recovery of BAP treatment in ALI-HBECs over time as measured by UPLC. A,B) BAP parent recovered summed over cell, media, and mucus fractions represented by percentage of BAP recovered in 0-hour samples for the optimization and validation datasets, respectively. C,D) BAP parent and Phase 1 metabolites recovered summed over cell, media, and mucus fractions represented by percentage of BAP recovered in 0-hour samples for the optimization and validation datasets, respectively. E) BAP parent and Phase 1 and 2 metabolites recovered summed over cell, media, and mucus fractions represented by percentage of BAP recovered in 0-hour samples for the validation dataset. Data bars represent the mean response at each timepoint. Error bars represent the standard error of the means.

ALI-HBECs were apically treated with BAP to assess parent and metabolite movement in the system. Vehicle control treated samples showed no evidence of background fluorescence at specified compound elution times. Sample extractions yielded an average extraction efficiency of 98.1 % ± 10.6 for the optimization dataset, and 84.7 % ± 19.8 for the validation dataset based on the DBC spike recovery. The average BAP recovery at 0 h was 85.2 % ± 0.8 (8.6 nmoles) for the optimization dataset and 87.5 % ± 7.0 (16.8 nmoles) for the validation dataset. This timepoint serves as a validation for the amount of BAP added during treatment and is used as the real amount of BAP added to the system when building the dosimetry model and calculating % BAP recovery (Fig. 2). When accounting for BAP parent only, BAP recovery remained consistent up to 4 h then decreased to 48 h in the optimization dataset (Fig. 2A), and a similar result was observed in the validation dataset (Fig. 2B). By 48 h, the amount of BAP parent was almost completely eliminated from the system in both datasets. Accounting for Phase 1 metabolites increases the BAP recovery, but the total recovery (BAP + metabolites) at any timepoint beyond 4 h fails to reach 100 % for both datasets (Fig. 2C and D). Accounting for BAP parent and Phase 1 and 2 metabolites significantly increases the amount of BAP recovered at 24 and 48 h when compared by *t*-test to the amount of BAP recovered from just BAP parent and Phase 1 metabolites at the same timepoints (Fig. 2E). The inability to recover the initial mass of BAP at all timepoints could be a result of chemical volatilization from the system, but most likely is due to an inability to quantify all possible metabolites. UPLC chromatograms show multiple unknown peaks that could not be identified from standards with a large peak eluting very close to 7,8-dihydrodiol (Figure S1).

**Fig. 3:**
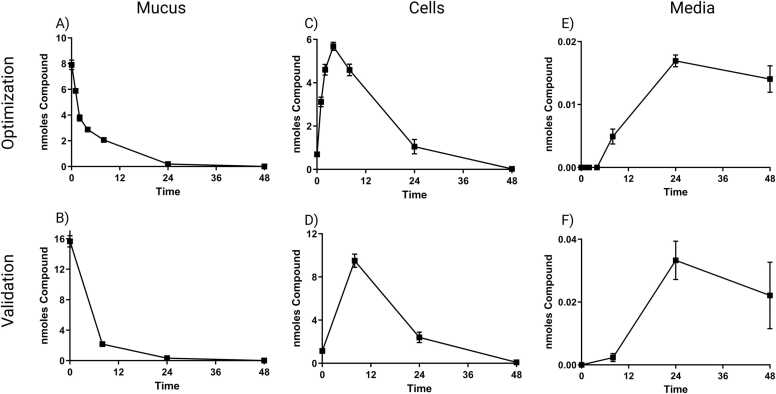
Amount of BAP parent in ALI-HBEC culture fractions over time as measured by UPLC. A,B) Amount of BAP parent in the mucus fraction, C,D) amount of BAP parent in the cell fraction, and E,F) amount of BAP parent in the media fraction for the optimization and validation datasets, respectively. Data points represent the mean response at each timepoint presented as the nmoles of BAP observed in that fraction. Error bars represent the standard error of the means.

BAP parent was observed to move throughout the culture system similarly in both datasets (Fig. 3). BAP was observed to leave the mucus fraction almost entirely by 48 h for both datasets (Fig. 3A and B). The optimization dataset demonstrates an early upward trend of BAP absorption into cells, and both datasets show a decrease of BAP from 8 to 48 h (Fig. 3C and D). The media fraction showed a similar trend in both experiments for the BAP parent with an increase in amount up to 24 h then a slight decrease to 48 h (Fig. 3E and F). The optimization dataset demonstrates that BAP did not start releasing into the media fraction until after 4 h. While BAP can be observed and measured in the media fraction, the amount of BAP reaching the media was very low in both datasets. The larger dose of BAP in the validation dataset resulted in more BAP in the cell and media fractions at similar timepoints but BAP disappearance from the mucus fraction appeared very similar between datasets from 8 to 48 h.

**Fig. 4:**
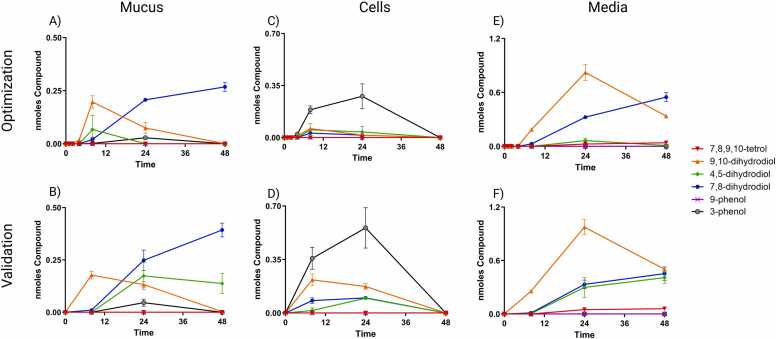
Amount of BAP Phase 1 metabolites in ALI-HBEC culture fractions over time as measured by UPLC. A,B) Amount of metabolites in the mucus fraction, C,D) amount of metabolites in the cell fraction, and E,F) amount of metabolites in the media fraction for the optimization and validation datasets, respectively. Data points represent the mean response at each timepoint presented as the nmoles of BAP metabolite observed in that fraction for each metabolite. Error bars represent the standard error of the means.

Phase 1 metabolites were observed to move similarly throughout the culture system in both datasets (Fig. 4). 9-phenol and 7,8,9,10-tetrol were considered minor metabolites due to their relatively low recovery in all fractions and timepoints. In mucus, there is an increase in all major metabolites between 8 and 24 h then a decrease to 48 h with the exception of 7,8-dihydrodiol which increases from 8 to 48 h (Fig. 4A and B). In cells, there is an increase in all major metabolites to 24 h then a decrease to 48 h for both datasets (Fig. 4C and D). In media, both datasets show an increase in all major metabolites except 3-phenol to 24 h then a decrease in all metabolites except 7,8-dihydrodiol and 7,8,9,10-tetrol to 48 h (Fig. 4E and F). 7,8-dihydrodiol and 7,8,9,10-tetrol continually increased in media over the full 48 h. The validation dataset showed a continual increase in 4,5-dihydrodiol in media from 8 to 48 h similar to the level of 7,8-dihydrodiol. The major differences between the datasets is the larger yield of 4,5-dihydrodiol in mucus and media fractions and a greater yield of all metabolites between 8 and 24 h in the cell fraction for the validation dataset. Metabolites were not observed until at least 4 h after treatment.

**Fig. 5:**
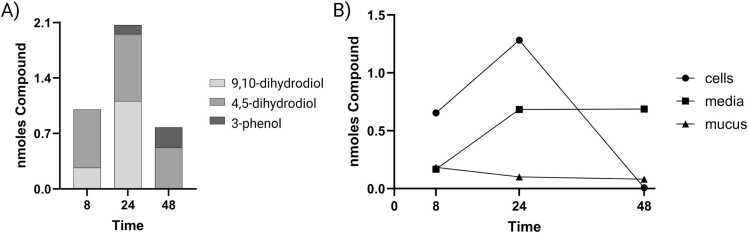
Amount of glucuronides in ALI-HBEC cultures over time as measured by UPLC. A) Average amount of glucuronides in the whole system presented as the deconjugated Phase 1 metabolite. Data bars represent the difference between the average amount of Phase 1 metabolite recovered between samples incubated with and without β-glucuronidase. B) Average amount of glucuronides in each culture fraction. Data points represent the difference between the average amount of Phase 1 metabolite recovered between samples incubated with and without β-glucuronidase in each fraction.

ALI-HBECs were able to form 3-phenol, 4,5-dihydrodiol, and 9,10-dihydrodiol-glucuronides with 4,5-dihydrodiol and 9,10-dihydrodiol-glucuronides being the major Phase 2 metabolites (Fig. 5A). 9,10-dihydrodiol-glucuronide was only observed before 48 h, and 3-phenol-glucuronide was only observed at 24 and 48 h. 4,5-dihydrodiol-glucuronide was the only glucuronide metabolite observed at every timepoint evaluated (8, 24, and 48 h). The total amount of glucuronides increased up to 24 h then decreased to 48 h. Glucuronides were observed mainly in the cell fraction at 8 and 24 h and in the media fraction at 24 and 48 h (Fig. 5B). Glucuronides were not found to excrete into the mucus fraction.

**Fig. 6:**
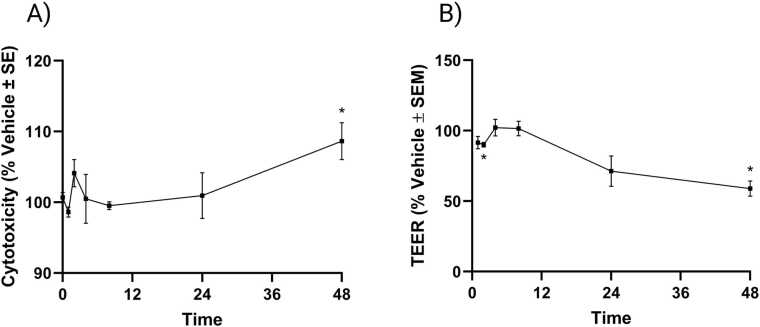
Cytotoxicity and barrier integrity after BAP exposure in ALI-HBECs evaluated by LDH leakage and TEER, respectively. A) Cytotoxicity as represented by the average % change normalized to the vehicle control. B) Barrier integrity as represented by the average % change normalized to the vehicle control. Error bars represent the standard error of the means. Significance was evaluated using a one-way ANOVA with Dunnett’s post-hoc test compared to the vehicle control (* p_adj_ < 0.05).

BAP treatment was found to significantly increase cytotoxicity (Fig. 6A) and significantly decrease barrier integrity (Fig. 6B) of ALI-HBECs at 48 h. These results were not considered when creating the dosimetry model due to evidence for most of the BAP parent being absorbed into the cells and metabolized or eliminated from the system by 48 h. Barrier integrity was also significantly decreased at 2 h, but this was not considered biologically relevant and did not appear to affect BAP parent or metabolite movement in the system.

### 3.2 Gene expression analysis

**Fig. 7:**
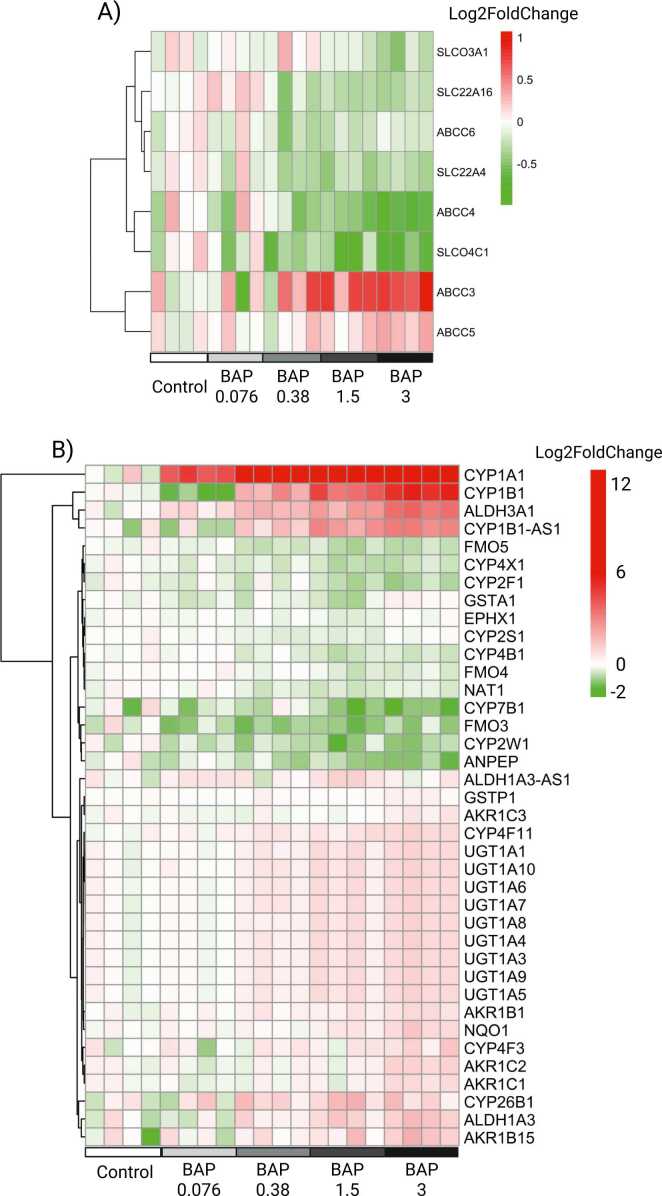
Differential expression of genes related to A) xenobiotic transport and B) metabolism evaluated by RNA-sequencing in BAP exposed ALI-HBECs. Values are Log2FoldChange where green, white, red represent a decrease, no change, and increase from vehicle controls, respectively. Significance of differential expression was evaluated by DESeq2 compared to vehicle control.

ALI-HBECs were evaluated for constitutive and differential gene expression after treatment with BAP for 24 h. This timepoint was chosen to correlate with the peak in metabolite formation. Xenobiotic metabolizing enzymes were identified to better understand observed metabolite formation, and xenobiotic transporters were identified for their potential role in metabolite distribution and excretion. ALI-HBECs constitutively expressed 18 genes related to xenobiotic transport (Table S1) consisting of genes in the organic anion/cation transporter (SLC22 and SLCO) and ATP-binding cassette subfamily C (ABCC) families and 81 genes related to xenobiotic metabolism (Table S2) consisting largely of genes in the cytochrome P450 (CYP450), aldehyde dehydrogenase (ALDH), UDP-glycosyltransferase (UGT), and glutathione S-transferase (GST) families. BAP exposure significantly differentially expressed 8 genes related to xenobiotic transport (Fig. 7A) by at least one treatment compared to vehicle control. All of the differentially expressed transporter genes were also constitutively expressed in controls. BAP exposure significantly differentially expressed 38 genes related to xenobiotic metabolism (Fig. 7B) by at least one treatment compared to vehicle control, and 33 of the 38 genes were also constitutively expressed in controls. The genes not constitutively expressed were *CYP1A1, CYP1B1-AS1, CYP7B1, CYP26B1*, and *AKR1B15*. *CYP1A1* had the largest magnitude Log2FoldChange after BAP exposure with an average induction of 12.6-fold and was the only gene significantly differentially expressed by all treatments of BAP.

### 3.3 CYP1A1 activity induction by BAP exposure

**Fig. 8:**
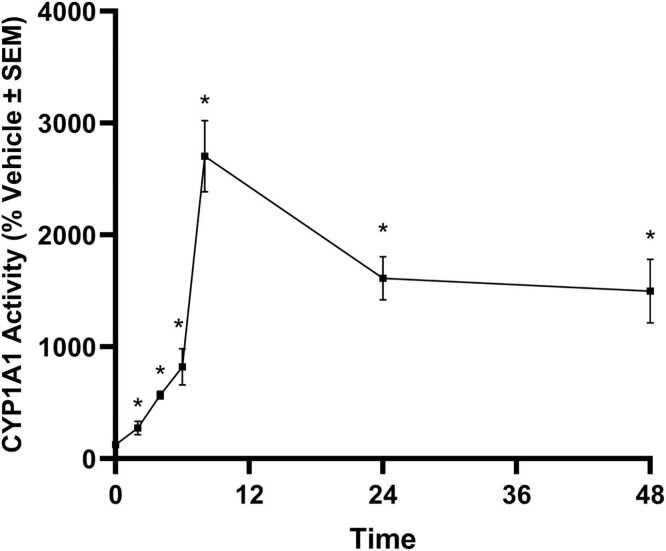
CYP1A1 activity over time for ALI-HBECs exposed to BAP evaluated by P450-Glo™ assay. Data points represent the mean response normalized to cell viability presented as the % change normalized to vehicle control. Error bars represent the standard error of the means. Significance was evaluated using a one-way ANOVA with Dunnett’s post-hoc test compared to the vehicle control (* p_adj_ < 0.05).

ALI-HBECs were evaluated for CYP1A1 enzyme activity after treatment with BAP (Fig. 8). BAP exposure significantly induced CYP1A1 activity at every timepoint tested after 0 h. Activity quickly increased reaching a maximum at 8 h then slowly decreased to 48 h.

### 3.4 Dosimetry model simulations

**Fig. 9:**
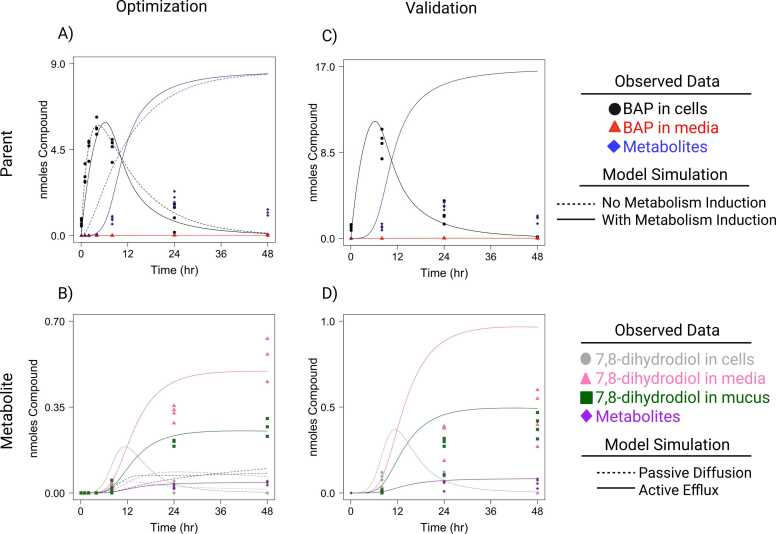
Cellular dosimetry model simulations compared to BAP and 7,8-dihydrodiol data collected by UPLC analysis over time. Model parameters were optimized on and simulations compared to data from the optimization dataset. A) Simulations for PAH parent compared to BAP data. Dash lines indicate model predictions without metabolism induction, and solid lines indicate model predictions with metabolism induction. B) Simulations for metabolite 1 compared to 7,8-dihydrodiol data. Dash lines indicate model predictions with passive diffusion out of cells, and solid lines indicate model predictions with active efflux into media and mucus compartments. C,D) Validation of model predictions with metabolism induction and active efflux compared to data from the validation dataset. Data points represent the amount of BAP in the cell and media fractions, total Phase 1 and 2 metabolites, 7,8-dihydrodiol in the cell, media, and mucus fractions, and total 7,8,9,10-tetrol as observed by UPLC.

A dosimetry model was constructed to describe PAH absorption, distribution, metabolism, and excretion (ADME) in ALI-HBECs. The model was optimized on BAP parent and metabolite data from the optimization UPLC dataset with and without the inclusion of metabolism induction (Fig. 9A). The model was able to describe the trends of BAP parent in the cell compartment within the error of the dataset. The model was able to describe the trends of BAP movement into the media compartment at 8, 24, and 48 h within the error of the dataset. The model overpredicted the amount of BAP in the media before 8 h where the dataset showed no BAP detected. However, the model prediction was relatively small on the order of 10^−5^. Since metabolites were not observed before 4 h, the model appeared to over-predict the amount of BAP metabolized at early timepoints (0 – 4 h). Therefore, we modeled the induction of metabolism as a 0–1 scaling factor using CYP1A1 activity data collected by P450-Glo™ assay. Several functions were considered to fit the data including polynomial, exponential, and hill functions. However, none of these functions were able to capture the dynamics of the CYP1A1 activity data which demonstrated a significant increase up to 8 h and a significant decrease from 8 to 48 h when evaluated by one-way ANOVA with Tukey’s post-hoc test (Figure S2). Ultimately, a rational function was fit to CYP1A1 activity data based on its ability to describe the dynamics of the data and the lowest AIC value of the attempted functions. Metabolism induction was included in the model for the amount of PAH parent metabolized, and the parameters were re-optimized on the optimization dataset. Including metabolism induction improved model predictions for the amount of BAP metabolized up to 8 h demonstrated by a decreased AIC from 75.8 to 45.2. The model predicts a large amount of BAP metabolism after 8 h in contrast to the observed Phase 1 metabolite formation. However, the observed data may be underestimating total metabolism due to an inability to quantify all metabolites especially as Phase 1 metabolites are further metabolized over time. Therefore, the observed Phase 1 metabolite data represent a minimum metabolism threshold, and the model exceeds this minimum.

To further understand the potential for toxic metabolite formation, the model also describes the ADME of a metabolite optimized on 7,8-dihydrodiol data from optimization UPLC dataset with passive diffusion or active efflux out of cells (Fig. 9B). The model describes the formation of 7,8-dihydrodiol in the cell compartment before 48 h within the error of the dataset. The model overpredicts the amount of 7,8-dihydrodiol in the cells at 48 h where no 7,8-dihydrodiol was detected. However, the model prediction was relatively small on the order of 10^−2^. The amount of 7,8-dihydrodiol metabolized is compared against the total amount of 7,8,9,10-tetrol observed in the optimization dataset representing a minimum metabolism threshold which the model exceeds and fits the trend of at early timepoints (before 4 h) where no 7,8,9,10-tetrol is detected. When only accounting for passive diffusion, the model underpredicts the amount of 7,8-dihydrodiol movement into media by 72 % at 24 h and 87 % at 48 h and the amount of 7,8-dihydrodiol in the mucus by 65 % at 24 h and 68 % at 48 h. Accounting for active efflux out of the cells greatly improves predictions and decreases the AIC for the amount of 7,8-dihydrodiol in media from 0.2 to −60.4 and the amount in mucus from −34.6 to −107.8.

The optimized model parameters demonstrate differences in kinetics for the parent and metabolite depending on model assumptions. Firstly, including metabolism induction affects the optimized value of all parameters, but the metabolism clearance terms had the greatest effect. BAP metabolism increased from 4.08 to 7.95 L/hr/kg^3/4^ demonstrating a maximal rate of metabolism once the model can scale the rate over time. When only accounting for passive diffusion, the model underpredicted metabolite distribution into media and mucus when permeability was assumed equal to the parent. Parent permeability was optimized at 6.93e^−5^ or 8.02e^−5^ cm/hr depending on metabolism induction while the metabolite was optimized at 0.061 cm/hr. The accumulation of 7,8-dihydrodiol in the media was the main driver of these differences and required a high rate of cell permeability to improve predictions, but even with the difference in permeability, the model still underpredicts 7,8-dihydrodiol movement into media and mucus. However, including active efflux of the metabolite into the media and mucus compartments allows the M1 permeability to be equal to the parent permeability and greatly improves the model’s predictions. Including active efflux also increases the fraction of 7,8-dihydrodiol formed from 0.022 to 0.099.

The model was validated by comparison to the validation UPLC dataset which was exposed to a higher dose of BAP (Fig. 9C and D). The model predicted BAP in the cells and media similar to the optimization dataset and exceeded the minimum metabolism threshold demonstrated by Phase 1 and 2 metabolite formation. The model predicted 7,8-dihydrodiol in the cells similar to the optimization dataset except overpredicted the amount at 8 h by 122 %. The model predicted the amount of 7,8-dihydrodiol in the mucus compartment within the error of the dataset except overpredicted the amount at 24 h by 53 %. The model exceeded the minimum metabolism threshold demonstrated by the 7,8,9,10-tetrol formation. However, the model over-predicted the amount of 7,8-dihydrodiol in the media by 16 %.

**Fig. 10:**
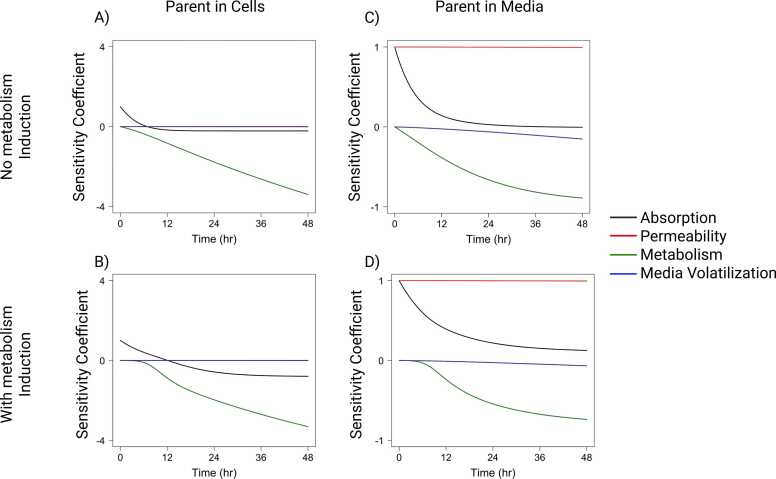
Sensitivity coefficients over time for model parameters with respect to PAH parent concentrations in A) cells without metabolism induction, B) cells with metabolism induction, C) media without metabolism induction, and D) media with metabolism induction.

**Fig. 11:**
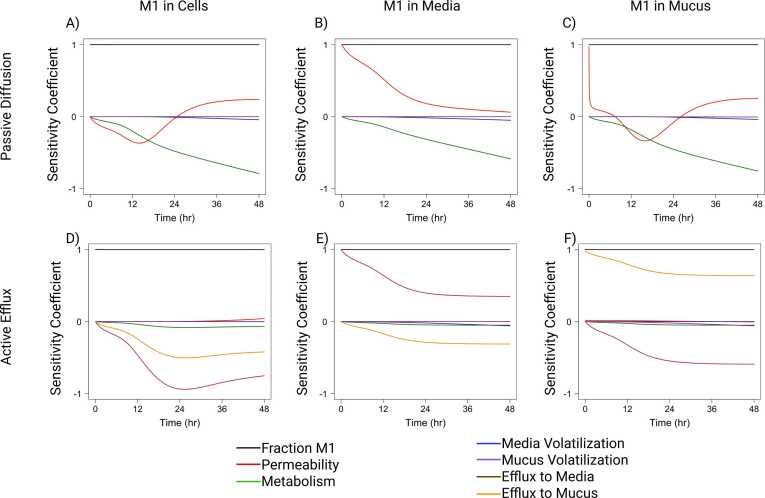
Sensitivity coefficients over time for model parameters with respect to metabolite (M1) concentrations in A) cells, B) media, and C) mucus with passive diffusion out of cells, and M1 concentrations in D) cells, E) media, and F) mucus with active efflux out of cells.

Sensitivity analysis indicates the importance of parameters for model simulations under given model assumptions. For parent concentration in cells, clearance by metabolism had low initial sensitivity which quickly increased over time resulting in the highest sensitivity for all parameters (Fig. 10A). Absorption into cells had the opposite trend of high sensitivity at early timepoints (before 8 h) which decreased quickly over time. This indicates that BAP absorption is the dominant pharmacokinetic process in early time points, while metabolism dominates later time points. Including metabolism induction decreased the sensitivity of metabolism before 8 h and slowed the decrease in sensitivity for absorption into cells (Fig. 10B). Permeability through cells had a very low sensitivity for parent concentration in cells. For parent concentration in media, permeability through cells had the highest sensitivity which stayed consistent over time both with and without metabolism induction (Fig. 10C and D). Clearance by metabolism and absorption into cells had similar trends as for parent concentration in cells, but metabolism had a much lower maximum sensitivity. Volatilization from media had a low sensitivity for parent concentration in cell and media compartments. For passive diffusion of M1, there was a similar trend of sensitivity for clearance by metabolism, media and mucus volatilization, and fraction of M1 formed with respect to the concentration of M1 in cells, media, and mucus compartments (Fig. 11A-C). The fraction of M1 formed from parent metabolism had the highest sensitivity which remained consistent over time. Similar to the parent, M1 metabolism sensitivity had low initial sensitivity which increased quickly over time. Media and mucus volatilization sensitivity were low for M1 concentration in all compartments. M1 permeability through cells had varying sensitivity over time with its highest sensitivity with respect to M1 concentration in mucus and media at early timepoints. When considering active efflux of M1, the trends for fraction of M1 formed and media and mucus volatilization sensitivity remained the same for M1 concentrations in all compartments as compared to the passive diffusion model (Fig. 11D-F). However, the sensitivity for clearance by metabolism and permeability of cells is much lower for M1 concentrations in all compartments. The initial sensitivity for active efflux into media and mucus is low and increases over time with respect to M1 concentration in cells. Active efflux into media sensitivity begins high and decreases over time for M1 concentration in media and has the opposite trend for M1 concentration in mucus. Active efflux into mucus had the opposite trend for M1 concentration in media and mucus compared to active efflux into media.

## 4 Discussion

There is a growing need for more human-relevant predictive models to aid in next generation risk assessment. These models can reduce time, effort, cost, and labor needed for toxicity testing by providing a reliable and human-relevant method of chemical prioritization. The similarity of ALI-HBEC morphology, cellular differentiation, and global constitutive mRNA expression to *in vivo* human lungs has already been explored, but the culture system has not yet been fully validated for its capacity to metabolize PAHs into toxic intermediates [24], [70]. Furthermore, it is important to understand the connection between nominal exposure concentration and internal tissue concentration for linking exposures to mechanistic outcomes. Understanding this connection expands the potential uses for NAMs for translating *in vitro* toxicity data for use in human health risk assessments. Presently, we explored the ability to utilize data collected in ALI-HBECs for predictive dosimetry modeling of PAH exposures and used the resulting model to aid in understanding PAH interactions with the system such as differential mRNA expression, PAH metabolism, and PAH and metabolite transport.

### 4.1 Xenobiotic metabolizing enzymes related to BAP metabolite formation in ALI-HBECs

In Phase 1 metabolism, BAP parent is mainly metabolized by CYP1A1 creating all of the Phase 1 metabolites observed in this study as well as many others [7], [52], [55]. Other CYP450s such as CYP1A2, CYP1B1, and CYP3A4 may also play a small role in the creation of these metabolites but to a much lesser extent [7], [73]. CYP1A1 and other CYP450s are the main metabolizers that create phenolic metabolites with 3-phenol and 9-phenol being the first and second most abundant, respectively [7], [33], [43], [71], [73], [80]. *CYP1A1* was not found to be constitutively expressed in ALI-HBECs but was very highly induced in both mRNA expression and protein activity with BAP exposure. BAP metabolite formation also correlated in time with CYP1A1 induction suggesting that CYP1A1 plays a large role in BAP metabolism in ALI-HBECs. *CYP1B1* was found to be constitutively expressed and highly induced from BAP exposure showing a potential for its role in BAP metabolism in ALI-HBECs as well. ALI-HBECs were observed to form a large amount of 3-phenol but only formed a very small amount of 9-phenol. CYP450s have also been shown to form epoxide metabolites which may be converted into dihydrodiols by microsomal epoxide hydrolases [4], [7], [31], [43], [73], [80], [92], [97]. 4,5-, 7,8-, and 9,10-dihydrodiol have been isolated as metabolites and are considered the major dihydrodiols formed from PAH metabolism [43], [92]. *EPHX1–3* were constitutively expressed in ALI-HBECs with *EPHX1* being highly constitutively expressed. Despite *EPHX1* being down-regulated by BAP exposure, ALI-HBECs were still able to form several dihydrodiol metabolites with 7,8- and 9,10-dihydrodiol as the major forms. Aldo-keto reductases (AKRs) have been shown to convert dihydrodiols into quinones with AKR1C1–3 being the most active and AKR1A1 being the major metabolizer of 7,8-dihydrodiol [73], [81], [97]. While we did not evaluate quinone formation in ALI-HBECs, we did observe several AKRs as constitutively expressed including *AKR1A1* and *AKR1C1–3* showing potential for ALI-HBECs to create quinone metabolites. Several AKRs were further induced by BAP exposure including *AKR1C1–3* but not *AKR1A1*. This could explain the continual increase in 7,8-dihydrodiol over time as compared to the other dihydrodiols which decrease between 24 and 48 h. Dihydrodiols may also be converted to diol epoxides by CYP450s. Specifically, the 7,8-dihydrodiol may be converted to BPDE by CYP1A1 which quickly degrades to 7,8,9,10-tetrol by hydrolysis [43], [55], [61], [80]. ALI-HBECs were observed to create the 7,8,9,10-tetrol metabolite suggesting the formation of BPDE as a cancerous intermediate of BAP metabolism in ALI-HBECs.

In Phase 2 metabolism, larger conjugates are added to Phase 1 metabolites to aid in detoxification and metabolite excretion by interactions with UGTs, sulfotransferases (SULTs), and GSTs. UGTs may convert Phase 1 metabolites into glucuronides with BAP- and 3-phenol-glucuronide being the major forms [43], [73], [80], [117]. ALI-HBECs were observed to constitutively express many UGTs, and BAP exposure further induced their expression. ALI-HBECs were observed to form several glucuronide metabolites including the 3-phenol form. However, the 4,5- and 9,10-dihydrodiol-glucuronide metabolites were the major metabolites observed. While the 7,8-dihydrodiol-glucuronide metabolite has been observed to form from isolated human liver microsomes, ALI-HBECs were not observed to form 7,8-dihydrodiol-glucuronide which could further explain the continual increase of 7,8-dihydrodiol over time compared to the other dihydrodiol metabolites which appear to be further metabolized by AKRs or UGTs [40], [99]. SULTs add a sulfate conjugate to Phase 1 metabolites with SULT1A1 being the major metabolizer [43], [80], [117], [120]. Sulfate metabolites can be deconjugated by β-glucuronidase incubation if sulfatase activity is not inhibited. We did not inhibit sulfatase activity which implies that our data could show evidence for deconjugated sulfates. While ALI-HBECs constitutively expressed a relatively high level of *SULT1A1*, BAP did not induce the expression of any SULTs and there were only 3 SULTs constitutively expressed compared to 10 constitutive UGTs and the induction of 9 UGTs after BAP exposure. This suggests that sulfate conjugation is not a major route of Phase 2 metabolism in ALI-HBECs especially when compared to glucuronide conjugation. Lastly, GSTs in the alpha, mu, and pi families and microsomal GSTs add a glutathione conjugate to Phase 1 metabolites [34], [43], [80]. We did not assess the formation of GST conjugates in ALI-HBECs. However, several GSTs were constitutively expressed, and BAP exposure either increased or decreased GST expression depending on the enzyme suggesting that glutathione metabolites may be formed in ALI-HBECs and requires further evaluation.

### 4.2 Modeling strategies for PAH dosimetry

Dosimetry, toxicokinetic, and pharmacokinetic models are used to assess the ADME of a chemical in a living system. Many models have been created for PAHs including BAP in several test systems such as rodents, dogs, and humans from various exposure routes such as intravenous, inhalation, and ingestion [19], [25], [26], [27], [32], [37], [44], [45], [46], [75], [79], [98], [109], [110], [111]. The presented model appears to be one of the first designed for describing PAH dosimetry in ALI-HBECs. These models can aid in understanding the relationship between exposure and internal dose and can be connected with health outcomes based on the predicted internal dose [63]. This has been demonstrated by correlating internal PAH doses with endpoints such as developmental toxicity, pathway activation, DNA adduct formation, and tumor incidence [25], [26], [45], [111]. Several studies have shown the adaptability of these models for other chemicals or systems. For example, Campbell et al. [19] converted a PBPK model developed for BAP exposures in rats to a PBPK model for BAP exposures in humans [19]. The resulting model was able to accurately predict BAP absorption and 3-phenol formation in humans. Another study by Chou et al. [26] was able to adapt a human PBPK model developed for nanomaterial exposure into a PBPK model for particulate matter (PM_2.5_)-bound PAH deposition in the lungs which could be correlated with pathway activation related to PAH exposures [26]. The presented dosimetry model may be adapted for other PAHs to understand differences in PAH kinetics, internal dosimetry, and potential for adverse health outcomes in ALI-HBECs. Only a handful of published models include the dosimetry of a metabolite, and they tend focus on the 3-phenol metabolite of BAP which has been linked with developmental toxicity [19], [37], [75], [111]. Here we evaluated the dosimetry of the 7,8-dihydrodiol metabolite known to be a precursor for BPDE, the major carcinogenic metabolite. Not including the dosimetry or at least the formation of toxic intermediates in the model could limit the model’s use for risk assessments of compounds that do not exert toxicity in the parent form. However, information on toxic metabolite dosimetry may be extrapolated from the internal dose of the parent if enough literature exists around the compound of interest.

Evaluating the parameters of dosimetry models can elucidate information on the kinetics for how compounds interact with a system. Published models on BAP dosimetry have reported a lung tissue:blood partitioning coefficient larger than 1 meaning that BAP parent preferentially stays in the tissue rather than excreting into the blood [19], [25], [37]. The presented model agrees with these kinetics having a lung cell:media partitioning coefficient of 110 as predicted by methods set by Poulin and Theil [85]. In models that include a metabolite, the 3-phenol metabolite is also reported to have a lung tissue:blood partitioning coefficient greater than 1 [19], [37]. The presented model evaluates the dosimetry of the 7,8-dihydrodiol metabolite which has a predicted lung cell:media partitioning coefficient of 73 and lung cell:mucus partitioning coefficient of 10.9 as predicted by methods set by Poulin and Theil [85] meaning that 7,8-dihydrodiol preferentially stays in the tissue rather than excreting into the media or mucus but to a lesser extent than the parent [85]. Evaluating the movement of the 3-phenol metabolite in ALI-HBECs shows that it tends to stay in the cell fraction and does not readily excrete into media similar to the published dosimetry models. This suggests that more polar metabolites will be more quickly excreted from cells when compared to the parent but ultimately prefer the tissue. Published models that include parent metabolism often use Michaelis-Menten (MM) kinetics to simulate the change in metabolism rate according to compound concentration [9], [19], [25], [27], [37], [79], [98]. We did not have the data necessary to inform a MM equation and instead modeled clearance by metabolism as a first order rate. However, the rate of metabolism appeared to be dynamic correlating with changes in *CYP1A1* expression and activity over time, and accounting for this dynamic metabolism rate improved the model’s predictions. The final model suggests 7,8-dihyodriol interactions with xenobiotic transporters for active efflux out of the cell compartment. We observed the constitutive and differential expression of several genes related to xenobiotic transport that supports the inclusion of active transport in the model [38]. While these genes are known to transport Phase 2 xenobiotic metabolites, there is a lack of knowledge surrounding the ability of these transporters to transport 7,8-dihydrodiol and requires further investigation. Evaluation of the model with the validation dataset shows good predictions for the parent in cells and amount metabolized, but the model overpredicts the amount of 7,8-dihydrodiol in media and mucus. When the model is temporarily optimized to the validation set, the average rate of active efflux is consistent with the optimization set. However, the fraction of M1 formed from parent metabolites is reduced from 0.099 to 0.05. Additionally, the Phase 1 metabolite profiles between the optimization and validation datasets show similar results for most metabolites except 4,5-dihydrodiol despite the higher concentration of BAP. From these results, it appears that CYP1A1, the main enzyme responsible for formation of the observed metabolites, is becoming saturated at the higher dose of BAP resulting in other xenobiotic metabolizing enzymes metabolizing more of the BAP parent. Several CYPs have been shown to differ in metabolite profiles such as CYP3A4 and CYP1A2 which preferentially make 4,5-dihydrodiol over 7,8-dihydrodiol [7]. This demonstrates the need to evaluate dosimetry models at multiple dosing concentrations to evaluate mechanistic differences important for compound ADME.

The presented model appears to well predict BAP parent and 7,8-dihydrodiol metabolite dosimetry in ALI-HBECs with the inclusion of metabolism induction and active efflux of the metabolite. However, there exist some limitations to the current model. Firstly, the model was optimized on exposures from high BAP doses and requires further validation for predictability at environmentally relevant exposure concentrations. Additionally, all experiments were conducted in cells from the same donor to simplify data analysis, but interindividual differences could play an important role in PAH dosimetry. Along with differences in gene expression of xenobiotic metabolizing enzymes as discussed above, several studies have shown the ADME of PAHs can change between individuals [8], [12], [93], [109]. Model parameters for metabolism as well as the induction of metabolism over time could also be donor dependent. If interindividual variation is found to be important for PAH ADME, for example with CYP1A1 polymorphisms, then the model can be adjusted to account for these variables. Furthermore, the model currently only simulates BAP dosimetry and may not reflect the kinetics of other PAHs. Additionally, environmental research is emphasizing the need for mixture toxicity assessments, and the model may require heavy adaptation to accurately simulate dosimetry for a PAH mixture due to potential for chemical interactions [14], [21]. A study by Smith et al. [98] demonstrated that PAHs may compete for enzymes when co-exposed, and a study by Vermillion-Maier et al. [66] showed potential for a PAH to inhibit enzymes necessary for the metabolism of another [66], [98]. These studies demonstrate that more complex toxicokinetics especially regarding metabolism may be necessary when adapting dosimetry models for PAH mixtures.

## 5 Conclusions

In this study, we built a cellular dosimetry model for PAH exposure in an ALI human lung model. This study helps demonstrate the usefulness of these systems for providing human-relevant and mechanistic toxicity data. We observed constitutive and BAP-induced mRNA expression of genes related to xenobiotic metabolism that were consistent with results previously reported in human lung tissues. ALI-HBECs expressed metabolizing enzymes necessary for known BAP metabolism pathways in human lungs, and several Phase 1 and 2 metabolites were observed to form over time. The cellular dosimetry model was able to well describe the ADME of the BAP parent and 7,8-dihydrodiol metabolite in cell, media, and mucus compartments over time and model parameters agreed with other published models for BAP in lung tissue. Overall, ALI-HBECs have been shown to be metabolically competent for PAH bioactivation, strongly mimic *in vivo* human lung morphology and functionality, and are a promising NAM for PAH toxicity assessments. The resulting dosimetry model can help inform extrapolation of toxicity observed *in vitro* to other systems, including correlating *in vitro* BAP toxicity results to BAP inhalation exposures and adverse health outcomes in humans. This model can also be further adapted for other PAHs and PAH mixtures to increase its use in PAH toxicity predictions, PAH prioritization, and IVIVE.

## CRediT authorship contribution statement

**Kelley M. Bastin:** Methodology, Investigation. **Lisbeth K. Siddens:** Writing – review & editing, Methodology. **Victoria C. Colvin:** Writing – review & editing, Writing – original draft, Visualization, Methodology, Investigation, Formal analysis, Data curation, Conceptualization. **Monica L. Vermillion Maier:** Writing – review & editing, Methodology. **David E. Williams:** Writing – review & editing, Supervision, Resources, Funding acquisition, Conceptualization. **Jordan N. Smith:** Writing – review & editing, Supervision, Methodology, Conceptualization. **Susan C. Tilton:** Writing – review & editing, Supervision, Funding acquisition, Data curation, Conceptualization.

## Funding

This project was supported by the 10.13039/100000066National Institute of Environmental Health Sciences under Award Numbers P42 ES016465, T32 ES007060 and P30 ES030287. This work is supported, in part, by the Oregon Agricultural Experiment Station with funding from the Hatch Act capacity funding program, award numbers NI25HFPXXXXXG022 and/or NI25HMFPXXXXG029, from the USDA National Institute of Food and Agriculture.

## Declaration of Competing Interest

The authors declare that they have no known competing financial interests or personal relationships that could have appeared to influence the work reported in this paper.

## Data Availability

Data will be made available on request.

## Footnotes

Appendix A Supplementary data associated with this article can be found in the online version at doi:10.1016/j.toxrep.2025.102133.
