# Supplementary material for: Building a predictive model for polycyclic aromatic hydrocarbon dosimetry in organotypically cultured human bronchial epithelial cells using benzo[a]pyrene
Source: Toxicol Rep. 2025 Sep 28;15:102133. doi: 10.1016/j.toxrep.2025.102133 (PMC12517105; doi:10.1016/j.toxrep.2025.102133)
Supplement: Supplementary file 1 — Supplementary material [file mmc1.docx]

**Supporting Information for Building a Predictive Model for PAH Dosimetry in ALI-HBECs using Benzo[a]pyrene**

Victoria C. Colvin^1,2^, Kelley M. Bastin^1,2^, Lisbeth K. Siddens^1,2^, Monica L. Vermillion Maier^1,2^, David E. Williams^1,2^, Jordan N. Smith^2,3^, Susan C. Tilton^1,2,*^

^1^ Department of Environmental and Molecular Toxicology, ^2^ OSU/PNNL Superfund Research Program, Oregon State University, Corvallis, OR, USA; ^3^ Pacific Northwest National Laboratory, Richland, WA, USA, ^*^correspondence: susan.tilton@oregonstate.edu

**Supplemental Tables and Legends**

**Table S1.** Constitutively expressed genes related to xenobiotic transportation in ALI-HBECs defined by FPKM>1 in at least one control sample.

| **Xenobiotic Transporters** | | | | | |
| --- | --- | --- | --- | --- | --- |
| **Gene** | **FPKM** | | | | **Average±STD** |
| ABCC1 | 15.46467 | 16.96964 | 17.47962 | 16.72683 | 16.66019±0.856531 |
| ABCC3 | 1.763768 | 1.220803 | 1.29416 | 1.328734 | 3.203867±0.240777 |
| ABCC4 | 1.447318 | 2.292355 | 1.859073 | 1.809576 | 1.401866±0.245429 |
| ABCC5 | 9.230695 | 7.596976 | 7.534473 | 8.841667 | 1.852081±0.346184 |
| ABCC6 | 4.763951 | 5.46888 | 5.510581 | 5.953006 | 8.300953±0.864073 |
| ABCC10 | 3.297132 | 3.458367 | 2.888595 | 3.171376 | 5.424105±0.491604 |
| SLC22A3 | 0.772351 | 1.223298 | 1.246804 | 0.505446 | 1.922099±0.227769 |
| SLC22A4 | 32.66022 | 36.84357 | 34.48132 | 36.64508 | 5.913323±0.255377 |
| SLC22A5 | 1.439246 | 1.458594 | 1.452293 | 1.422174 | 0.876635±0.149279 |
| SLC22A15 | 2.026201 | 2.13375 | 1.922578 | 1.605868 | 3.294867±0.685681 |
| SLC22A16 | 5.832297 | 5.708275 | 5.826008 | 6.286711 | 17.47173±0.333564 |
| SLC22A17 | 0.91336 | 0.661784 | 0.923993 | 1.007402 | 0.936975±0.361153 |
| SLC22A18 | 4.188562 | 2.518192 | 3.265628 | 3.207086 | 35.15755±1.979002 |
| SLC22A23 | 17.07467 | 17.8017 | 17.68557 | 17.32498 | 1.443076±0.016097 |
| SLCO1B3 | 1.113458 | 1.178294 | 1.273034 | 1.214457 | 1.194811±0.066823 |
| SLCO3A1 | 2.590549 | 3.206806 | 2.977257 | 2.588634 | 2.840812±0.304846 |
| SLCO4A1-AS2 | 1.285279 | 0.962349 | 1.031729 | 1.309288 | 1.147161±0.175918 |
| SLCO4C1 | 3.146725 | 4.013789 | 3.925958 | 4.531581 | 3.904513±0.571506 |

**Table S2.** Constitutively expressed genes related to xenobiotic metabolism in ALI-HBECs defined by FPKM>1 in at least one control sample.

| **Xenobiotic Metabolizing Enzymes** | | | | | |
| --- | --- | --- | --- | --- | --- |
| **Gene** | **FPKM** | | | | **Average±STD** |
| AKR1A1 | 30.62257 | 31.70366 | 30.02038 | 32.62385 | 31.24262±1.154513 |
| AKR1B1 | 7.255486 | 9.125523 | 6.371783 | 6.357055 | 7.277462±1.301694 |
| AKR1B10 | 10.89957 | 19.10116 | 8.167571 | 5.507416 | 10.91893±5.882281 |
| AKR1C1 | 22.79778 | 22.80594 | 18.95522 | 18.8407 | 20.84991±2.254408 |
| AKR1C2 | 30.79548 | 28.6875 | 24.38542 | 22.43395 | 26.57559±3.839171 |
| AKR1C3 | 18.89076 | 23.0543 | 19.23388 | 18.40089 | 19.89496±2.133784 |
| AKR7A2 | 18.78699 | 18.47622 | 17.67535 | 19.57673 | 18.62882±0.786539 |
| ALDH1A1 | 224.7202 | 211.4619 | 239.5147 | 292.3275 | 9.162384±0.47712 |
| ALDH1A3 | 36.76257 | 76.4969 | 46.17968 | 30.3309 | 23.70585±1.010011 |
| ALDH1A3-AS1 | 1.045815 | 0.745734 | 0.867671 | 0.598856 | 242.0061±35.45047 |
| ALDH1B1 | 3.247543 | 3.249751 | 3.0045 | 3.146508 | 47.44251±20.4338 |
| ALDH1L1 | 2.000568 | 1.994256 | 1.960406 | 1.995203 | 0.814519±0.189354 |
| ALDH2 | 19.1589 | 19.52156 | 19.11204 | 19.33356 | 3.162075±0.115563 |
| ALDH3A1 | 64.32993 | 38.17159 | 52.88208 | 53.72018 | 1.987608±0.018346 |
| ALDH3A2 | 10.55859 | 8.569489 | 10.07063 | 10.39557 | 19.28151±0.186266 |
| ALDH3B1 | 78.99909 | 90.25052 | 85.20076 | 87.05851 | 52.27594±10.74995 |
| ALDH3B2 | 7.362176 | 6.520518 | 6.827432 | 7.548981 | 9.898572±0.908975 |
| ALDH4A1 | 7.561605 | 9.326558 | 6.587564 | 6.187388 | 85.37722±4.735942 |
| ALDH5A1 | 5.770918 | 5.610308 | 5.847379 | 6.165467 | 7.064777±0.4745 |
| ALDH6A1 | 3.082273 | 2.303179 | 2.764129 | 2.993486 | 7.415779±1.398478 |
| ALDH7A1 | 3.551843 | 3.467932 | 3.21411 | 3.640302 | 5.848518±0.233255 |
| ALDH9A1 | 30.67597 | 33.81643 | 30.72594 | 33.3886 | 2.785767±0.348531 |
| ALDH16A1 | 9.424944 | 9.681972 | 8.909408 | 8.633211 | 3.468547±0.183645 |
| ALDH18A1 | 22.51073 | 23.25791 | 24.32537 | 24.72938 | 32.15174±1.684418 |
| ANPEP | 3.065541 | 3.937313 | 5.370169 | 2.514812 | 3.721959±1.245132 |
| CYP1B1 | 3.542613 | 3.998119 | 3.087646 | 2.999129 | 3.406877±0.460492 |
| CYP2A13 | 1.917157 | 2.618329 | 3.128573 | 3.959434 | 1.893433±0.146954 |
| CYP2A6 | 1.675557 | 2.19033 | 2.008875 | 2.264004 | 3.848305±2.649961 |
| CYP2B6 | 0.974316 | 1.292695 | 1.149827 | 1.163842 | 6.088116±1.101867 |
| CYP2C18 | 3.964734 | 2.578336 | 3.434744 | 3.873309 | 6.65534±1.458353 |
| CYP2F1 | 46.92271 | 68.35365 | 57.46298 | 48.66292 | 1.235104±0.205091 |
| CYP2J2 | 3.493278 | 4.708601 | 4.112533 | 3.617228 | 2.905873±0.860199 |
| CYP2R1 | 1.403823 | 1.054212 | 0.983843 | 1.18665 | 2.034691±0.26233 |
| CYP2S1 | 42.23783 | 40.95831 | 40.84757 | 44.9663 | 1.14517±0.130799 |
| CYP2U1 | 1.080714 | 1.542157 | 1.296304 | 1.257058 | 3.462781±0.633382 |

**Table S2 (Continued).** Constitutively expressed genes related to xenobiotic metabolism in ALI-HBECs defined by FPKM>1 in at least one control sample.

| **Xenobiotic Metabolizing Enzymes** | | | | | |
| --- | --- | --- | --- | --- | --- |
| **Gene** | **FPKM** | | | | **Average±STD** |
| CYP2W1 | 4.207796 | 2.492247 | 3.666126 | 4.00723 | 55.35056±9.81998 |
| CYP4B1 | 169.4807 | 162.3839 | 168.8889 | 188.4922 | 3.98291±0.552839 |
| CYP4F11 | 3.623299 | 3.688007 | 3.550003 | 4.503696 | 1.157132±0.184707 |
| CYP4F12 | 1.480779 | 0.811403 | 0.911426 | 0.887365 | 42.2525±1.916046 |
| CYP4F3 | 3.296285 | 1.899088 | 2.444064 | 2.580808 | 1.294058±0.190125 |
| CYP4V2 | 3.530964 | 4.504538 | 3.786544 | 3.884527 | 3.59335±0.767369 |
| CYP4X1 | 30.17489 | 39.81428 | 31.25502 | 29.69111 | 3.710105±0.455827 |
| CYP4Z1 | 0.934012 | 1.061757 | 0.808604 | 0.59214 | 172.3114±11.25612 |
| CYP20A1 | 1.993165 | 2.034716 | 1.718912 | 1.826937 | 3.841251±0.445214 |
| CYP24A1 | 3.285457 | 7.696274 | 2.728813 | 1.682675 | 1.022743±0.308318 |
| CYP26A1 | 6.475534 | 5.866955 | 4.695502 | 7.314474 | 2.555061±0.575238 |
| CYP27A1 | 5.464738 | 8.30716 | 7.45623 | 5.393232 | 3.926643±0.413089 |
| CYP27C1 | 1.220751 | 1.394847 | 1.374417 | 0.950403 | 32.73382±4.76536 |
| CYP39A1 | 3.112949 | 4.080504 | 3.595629 | 4.051337 | 0.849128±0.200085 |
| CYP51A1 | 19.57277 | 21.48171 | 19.87481 | 23.01891 | 20.98705±1.592734 |
| EPHX1 | 87.28751 | 96.46482 | 91.70376 | 97.2205 | 93.16915±4.619376 |
| EPHX2 | 8.607084 | 7.749512 | 8.17404 | 9.189097 | 8.429933±0.615404 |
| EPHX3 | 13.27448 | 13.99655 | 12.77433 | 9.673371 | 12.42968±1.904801 |
| FMO2 | 0.593605 | 1.226895 | 0.89496 | 1.005859 | 0.930329±0.263496 |
| FMO3 | 0.840991 | 1.921509 | 0.94555 | 1.175781 | 1.220958±0.487523 |
| FMO4 | 3.913182 | 4.3953 | 4.229912 | 4.501289 | 4.259921±0.256719 |
| FMO5 | 2.746119 | 2.629953 | 2.477934 | 3.500807 | 2.838703±0.454857 |
| GSTA1 | 108.7879 | 115.5751 | 96.26402 | 115.7548 | 109.0955±9.148276 |
| GSTA2 | 6.639506 | 4.664301 | 5.113309 | 10.78044 | 6.799389±2.785427 |
| GSTA3 | 7.9021 | 11.11798 | 9.334699 | 9.590469 | 9.486311±1.317306 |
| GSTA4 | 15.3091 | 11.40136 | 13.22779 | 15.49889 | 13.85928±1.934811 |
| GSTM4 | 1.22816 | 0.917896 | 1.166545 | 1.245794 | 1.139599±0.151656 |
| GSTP1 | 441.0145 | 435.5793 | 431.4031 | 470.4456 | 444.6106±17.66714 |
| MGST1 | 20.32915 | 22.45862 | 19.59525 | 21.8231 | 21.05153±1.318814 |
| MGST2 | 10.20918 | 9.374625 | 10.01904 | 10.37431 | 9.994287±0.437869 |
| MGST3 | 7.624547 | 7.871993 | 8.098401 | 8.23246 | 7.95685±0.266848 |
| NAT1 | 13.2352 | 16.20625 | 15.1583 | 15.63973 | 15.05987±1.289644 |
| NQO1 | 53.37841 | 64.8527 | 54.88528 | 52.86156 | 56.49449±5.63789 |
| SULT1A1 | 21.91037 | 21.83984 | 20.96081 | 27.40622 | 23.02931±2.94974 |
| SULT1E1 | 2.397104 | 2.127813 | 1.664361 | 3.302578 | 2.372964±0.689682 |

**Table S2 (Continued).** Constitutively expressed genes related to xenobiotic metabolism in ALI-HBECs defined by FPKM>1 in at least one control sample.

| **Xenobiotic Metabolizing Enzymes** | | | | | |
| --- | --- | --- | --- | --- | --- |
| **Gene** | **FPKM** | | | | **Average±STD** |
| SULT2B1 | 3.859441 | 3.334347 | 3.2776 | 5.019857 | 3.872811±0.808314 |
| UGT1A1 | 7.875117 | 7.646598 | 6.367939 | 6.811362 | 7.175254±0.706198 |
| UGT1A3 | 14.13223 | 13.66279 | 11.48566 | 12.20942 | 9.998563±0.962508 |
| UGT1A4 | 13.42811 | 12.96597 | 10.91944 | 11.60512 | 12.87253±1.234839 |
| UGT1A5 | 14.13223 | 13.64491 | 11.48566 | 12.20942 | 12.22966±1.166929 |
| UGT1A6 | 8.125025 | 7.917419 | 6.760804 | 7.141536 | 12.86805±1.23105 |
| UGT1A7 | 8.837096 | 8.570763 | 7.073157 | 7.621159 | 7.486196±0.642661 |
| UGT1A8 | 14.42501 | 13.8778 | 11.69854 | 12.49948 | 8.025544±0.821877 |
| UGT1A9 | 14.48986 | 13.9902 | 11.77632 | 12.51839 | 13.12521±1.24937 |
| UGT1A10 | 10.96212 | 10.64139 | 8.923936 | 9.466805 | 13.19369±1.262203 |
| UGT2A2 | 0.614309 | 0.42323 | 0.422085 | 1.105553 | 0.641294±0.322423 |


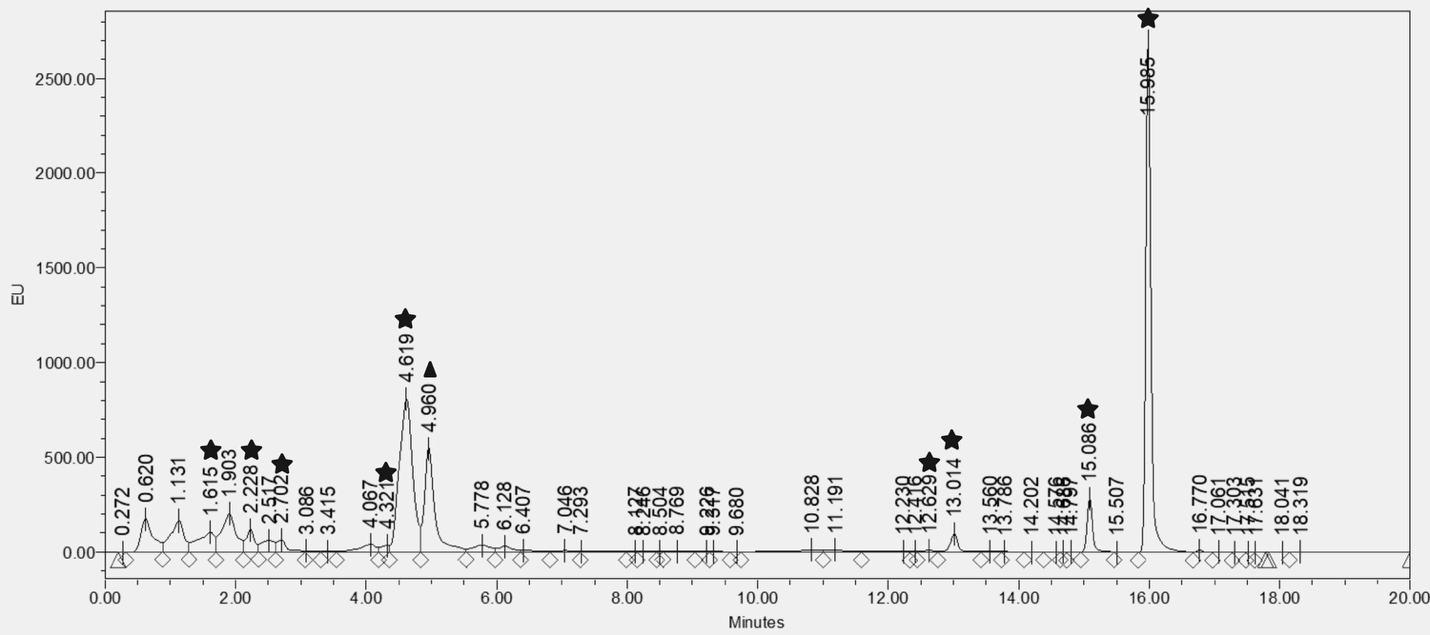


**Figure S1.** Representative chromatogram for the media fraction of a BAP treated sample at 24 hours post exposure. Stars indicate known BAP parent, Phase 1 metabolites, and DBC internal standard. Triangle indicates a major unknown metabolite.


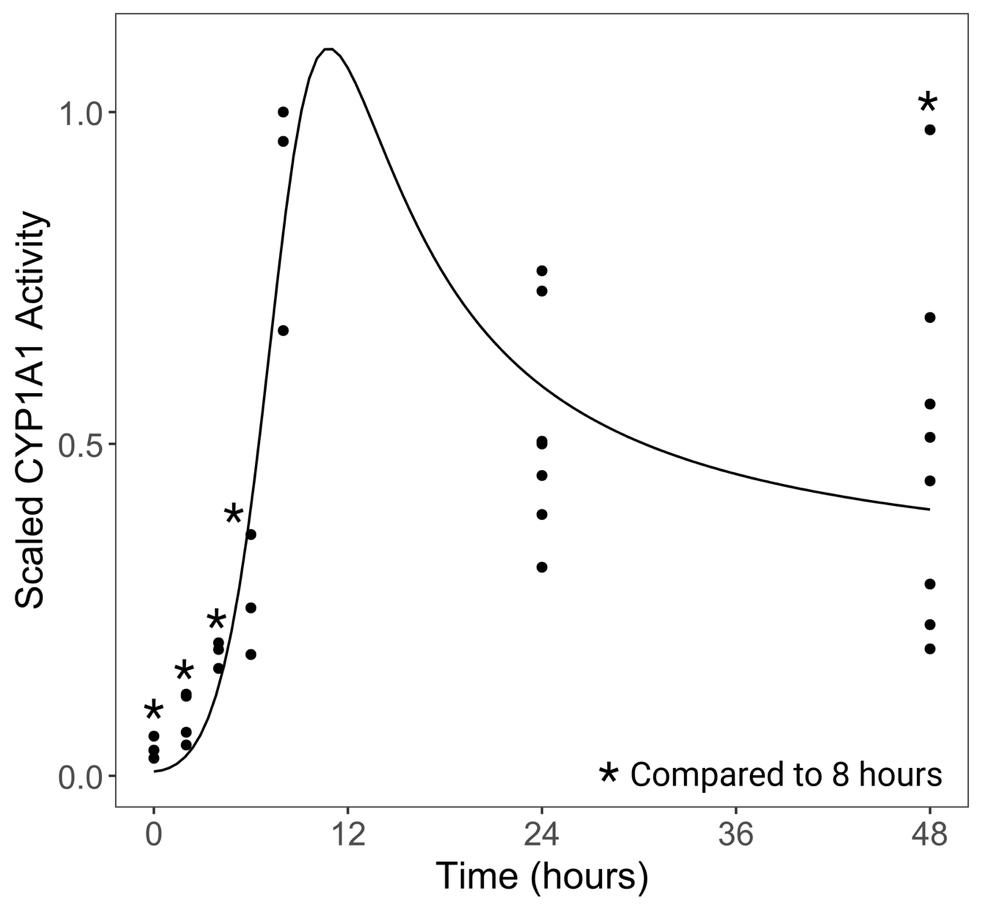


**Figure S2.** Rational function fit to CYP1A1 activity data evaluated by P450-Glo™ assay. CYP1A1 activity in each sample was normalized to cell viability evaluated by CTG assay, normalized to vehicle control at the respective timepoint, and scaled between 0 and 1 where 1 is the max response observed in the data. Significance was evaluated using a one-way ANOVA with Tukey’s post-hoc test compared to the 8-hour treatment group (* p_adj_ < 0.05).
